# Statistical Methodological Issues in Handling of Fatty Acid Data: Percentage or Concentration, Imputation and Indices

**DOI:** 10.1007/s11745-012-3665-2

**Published:** 2012-03-25

**Authors:** Roel J. T. Mocking, Johanna Assies, Anja Lok, Henricus G. Ruhé, Maarten W. J. Koeter, Ieke Visser, Claudi L. H. Bockting, Aart H. Schene

**Affiliations:** 1 Department of Psychiatry, Academic Medical Center, University of Amsterdam, Meibergdreef 5, Amsterdam, 1105 AZ The Netherlands; 2 Department of Clinical Psychology, University of Groningen, Grote Kruisstraat 2/1, Groningen, 9712 TS The Netherlands

**Keywords:** Multiple imputation, Non-detectable values, Undetectable, Peroxidation index (PI), Unsaturation index (UI), Chain length index, Eicosapentaenoic acid (EPA), Docosahexaenoic acid (DHA), Polyunsaturated fatty acids (PUFA), Recurrent major depressive disorder

## Abstract

Basic aspects in the handling of fatty acid-data have remained largely underexposed. Of these, we aimed to address three statistical methodological issues, by quantitatively exemplifying their imminent confounding impact on analytical outcomes: (1) presenting results as relative percentages or absolute concentrations, (2) handling of missing/non-detectable values, and (3) using structural indices for data-reduction. Therefore, we reanalyzed an example dataset containing erythrocyte fatty acid-concentrations of 137 recurrently depressed patients and 73 controls. First, correlations between data presented as percentages and concentrations varied for different fatty acids, depending on their correlation with the total fatty acid-concentration. Second, multiple imputation of non-detects resulted in differences in significance compared to zero-substitution or omission of non-detects. Third, patients’ chain length-, unsaturation-, and peroxidation-indices were significantly lower compared to controls, which corresponded with patterns interpreted from individual fatty acid tests. In conclusion, results from our example dataset show that statistical methodological choices can have a significant influence on outcomes of fatty acid analysis, which emphasizes the relevance of: (1) hypothesis-based fatty acid-presentation (percentages or concentrations), (2) multiple imputation, preventing bias introduced by non-detects; and (3) the possibility of using (structural) indices, to delineate fatty acid-patterns thereby preventing multiple testing.

## Introduction

Clinical fatty acid (FA)-research is becoming increasingly performed, but basic statistical methodological issues have remained largely underexposed in scientific literature thus far. We aim to address three of these issues in the handling of FA-data, and provide quantitative examples of their imminent confounding impact on results of FA-analyses, which may confuse the understanding of the roles FA play in (patho)physiology.

First, FA are reported in two ways: as absolute concentrations, or as percentages of the total FA-concentration. The implications of these different presentations have been scarcely addressed. Importantly, the few studies that have investigated this question showed significant differences between both approaches [1–3]. This is conceivable, because an increase in the percentage of one FA automatically results in the decrease in the relative percentage of another FA, even when its absolute concentration remains unchanged [4, 5]. Nevertheless, recent research still seems to opt rather randomly for either presentational method.

A second methodological issue is how to handle non-detectable FA-concentrations. In contrast to other research fields [6], FA-research thus far has not addressed this problem. Therefore, possible important analytical consequences remain uninvestigated, which may potentially cause biases in the interpretation of FA-data.

Third, because of the great number and variety of FA, the risk exists that multiple testing induces type-I errors, or the need for strict correction [7, 8]. A solution to this problem could be meaningful data-reduction, decreasing the number of tests needed. One possible way to achieve data-reduction might be the use of indices, delineating distinct FA-patterns by incorporating several FA-concentrations into one variable [9]. Examples of important patterns in FA-research are chain length, unsaturation, and peroxidizability of FA, because these characteristics modulate membrane fluidity and susceptibility to radical attack and are thereby conceivably involved in the pathogenesis of e.g. recurrent depression [10, 11]. Using indices, e.g. the unsaturation index (UI), chain length index (CLI) or peroxidation index (PI) [9], would allow testing of these more complex hypotheses on FA-patterns involving multiple FA, thereby obviating the need to interpret analyses of every individual FA to test your hypothesis. Thus far, most FA-research did not correct for multiple testing [7], and tested indices only in addition to the individual FA. This might potentially have resulted in type-I errors, and thereby bias in the interpretation of FA-analyses.

In this paper, the conceivable confounding effects of these three statistical methodological issues are examined, by providing quantitative examples in a practical research setting, using an example dataset of FA-concentrations of recurrently depressed patient and healthy controls, described previously [10]. This was done on the basis of the following research questions: (1) what is the influence of presentation of results in percentages or concentrations, and how does this differ for different FA, (2) what is the influence of the approach used for missing/non-detectable FA-concentrations on the significances of outcome differences; and (3) what is the influence of the use of indices for data-reduction on outcome differences?

## Materials and Methods

To investigate our research questions, we reanalyzed an example dataset consisting of washed erythrocyte FA-concentrations (pmol/10^6^ erythrocytes) from 137 recurrently depressed patients and 73 age- and sex-matched controls, determined by capillary gas chromatography, described in more detail previously [10, 12, 13].

### Percentages or Concentrations

To investigate the effects of presentational method, we expressed FA-concentrations both in concentrations (pmol/10^6^ erythrocytes) and molecular percentages (individual FA’s concentrations divided by the total FA-concentration). Subsequently, to quantify the difference between the two presentational methods for each FA, we calculated the correlation between its presentation as a percentage or as a concentration using Pearson’s *r* (*r*_absolute–percentual_; Table 1). A *r*_absolute–percentual_ of 1.00 (perfect correlation) indicates no difference between the two types of FA presentation, while a *r*_absolute–percentual_ closer to zero indicates larger differences.

**Table 1:** Effects of method of presentation (percentages or concentrations) and handling of non-detectable/missing values on fatty acid (FA) results in example dataset of 137 recurrently depressed patients and 73 non-depressed controls

Presentational effects				Non-detectable/missing values					
				Zero substitution^4^		Omission^5^		Imputation^6^	
FA	*r*_absolute–percentual_^1^	Mean^2^	|*r*|_FA-total_^3^	Patients	Controls	Patients	Controls	Patients	Controls
18:3n-3	0.96	0.83	0.38	0.84	0.81	0.85	0.83	0.83	0.81
18:4n-3	0.99	0.19	0.01	0.21^7^	0.035	0.45	0.31	0.24^7^	0.08
20:5n-3	0.97	3.55	0.21	3.3	3.9	3.4	3.9	3.3^9^	3.9
22:5n-3	0.91	8.87	0.37	7.9^7^	10.5	7.9^7^	10.5	8.0^7^	10.6
22:6n-3	0.95	16.75	0.29	14.8^7^	20.1	14.8^7^	20.1	14.9^7^	20.2
18:2n-6	0.85	66.26	0.66	66	67	66	67	66	67
18:3n-6	0.98	0.52	0.10	0.57^7^	0.38	0.61	0.62	0.58^7^	0.41
20:3n-6	0.92	9.23	0.37	8.8^9^	9.7	8.8^9^	9.7	8.9^8^	9.8
20:4n-6	0.62	75.22	0.72	71.5^7^	81.6	72.1^7^	81.6	72.0^7^	81.3
22:4n-6	0.91	11.45	0.22	10.7^7^	13.0	10.7^7^	13.0	10.6^7^	13.0
22:5n-6	0.95	1.85	0.14	1.7^7^	2.1	1.7^7^	2.1	1.7^7^	2.1
20:2n-6	0.95	1.33	0.23	1.3	1.3	1.4	1.3	1.3	1.3
22:2n-6	0.99	0.43	0.06	0.37^7^	0	0.79	ND	0.51^7^	0.26
14:1n-5	0.99	0.42	0.07	0.25^7^	0.60	0.57^7^	1.15	0.30^7^	0.65
16:1n-7	0.98	2.95	0.34	3.0^9^	2.5	3.0^9^	2.5	3.1^9^	2.6
18:1n-7	0.86	7.63	0.72	7.5	7.9	7.6	7.9	7.5	7.9
20:1n-7	0.99	0.29	0.14	0.21	0.27	0.66^7^	0.46	0.27	0.34
16:1n-9	1.00	1.27	0.19	0.9^9^	1.9	0.9^8^	1.9	0.93^8^	1.19
18:1n-9	0.69	74.78	0.79	74	75	75	75	75	75
20:1n-9	0.94	1.21	0.31	1.2	1.2	1.2	1.3	1.2	1.2
22:1n-9	0.99	1.96	0.03	1.9	2.1	3.1^8^	2.2	1.9	2.1
24:1n-9	0.93	15.47	0.29	13.3^7^	19.6	13.3^7^	19.6	13.3^7^	19.5
20:3n-9	0.99	0.36	0.04	0.36	0.31	0.42	0.40	0.38	0.32
14:0	0.95	3.34	0.34	3.3	3.5	3.3	3.5	3.3	3.5
16:0	0.70	160.7	0.78	164^9^	156	164^9^	156	163^9^	156
18:0	0.30	103.8	0.87	103	104	103	105	103	105
20:0	0.76	2.63	0.59	2.5^7^	2.8	2.6^7^	2.8	2.5^7^	2.8
22:0	0.88	8.26	0.22	7.6^7^	9.5	7.6^7^	9.5	7.6^7^	9.5
24:0	0.93	17.06	0.23	14.8^7^	21.3	14.7^7^	21.3	14.9^7^	21.2

All correlations were calculated using Pearson’s *r*

^1^Correlations between results presented in absolute concentrations (pmol/10^6^ erythrocytes) or relative percentages (FA_(*i*)_ concentration divided by the total FA-concentration). All correlations were significant with *P* < 0.001

^2^Mean concentrations (pmol/10^6^ erythrocytes)

^3^Modulus of the correlation between mean FA_(*i*)_ concentration and total FA concentration

Differences in concentrations between patients and controls, calculated using independent Student's *t* tests, after: ^4^ substitution of non-detectable values with zero, ^5^ omission of non-detects, and ^6^ multiple imputation of missing and non-detectable values

Significant compared to controls with ^7^ *P* < 0.001, ^8^ *P* < 0.01, and ^9^ *P* < 0.05

To learn how, for individual FA, presentation as concentrations or percentages results in differential biases, we investigated whether the difference between the two types of presentation (expressed as *r*_absolute–percentual_) depended on individual FA-characteristics. Therefore, we performed a second-level analysis exploring the relation between characteristics of the different individual FA and their observed *r*_absolute–percentual_. We first calculated each FA’s mean concentration (mean_FA(*i*)_; Table 1). Subsequently, for each FA, we calculated the absolute value (non-negative) of the correlation between the specific FA-concentration and the total FA-concentration for an individual subject (|*r*|_FA(*i*)-concentration–FA-total_; Table 1). Finally, we determined the influence of these individual FA-characteristics (mean_FA(*i*)_ and |*r*|_FA(*i*)-concentration–FA-total_) by entering these in a stepwise linear regression model as predicting variables with *r*(*i*)_absolute–percentual_ (after Fisher *r*-to-*Z* transformation [14]) as dependent variable.

### Handling of Non-detectable Values

To examine the influence of the handling of non-detectable/missing values, we compared: (1) substituting non-detectable values with zero, and omitting missing values; (2) omitting both non-detectable and missing values; and (3) using multiple imputation (MI) to estimate both non-detectable and missing values, using the software package Amelia II [15]. Simulation research previously demonstrated that MI was able to provide highly valid estimations of non-measured values, while incorporating the uncertainty involved [6, 16]. MI has been used on missing FA-concentrations before [17, 18], but not on non-detectable FA-concentrations.

To impute non-detectable/missing values, we used information on sex, age, marital status, educational level, social class, Hamilton Depression Rating Scale score, weight, length, waist and hip circumference, smoking, and salivary cortisol and dehydroepiandrosterone sulphate, folic acid, vitamin B_6_ and B_12_, homocysteine, and all other measured FA-concentrations. In addition, for non-detectable values, we assigned range priors in Amelia II indicating that a non-detectable FA concentration must lie between 0.001 and the detection limit of that FA (99 % confidence).

We used differences in erythrocyte FA-concentrations between patients and controls as example outcomes, calculated with independent Student's *t* tests. We compared the results of these different approaches to handle non-detectable/missing values to demonstrate their impact.

### Calculation of Indices

To investigate the influence of the use of indices on outcome differences we compared two methods. First, we compared the 29 individual FA concentrations in our example dataset between patients and controls using Student's *t* tests and a Bonferroni correction. We interpreted the outcome differences to detect patterns of differences in chain length, unsaturation or peroxidizability between patients and controls.

As an alternative to the interpretation of these multiple individual FA-tests, we applied data-reduction using indices, which we compared between patients and controls using Student's *t* tests. We selected three indices specifically designed to delineate patterns in chain length, unsaturation or peroxidizability.

1. The chain length index (CLI), providing information about FA-chain length. We calculated the CLI by adding the products of each FA’s concentration and the number of carbon atoms in their carbon chain and dividing this with the total FA-concentration;
2. The unsaturation index (UI), indicating the number of double bounds per FA. Calculated as follows: (1 × monoenoics + 2 × dienoics + 3 × trienoics + 4 × tetraenoics + 5 × pentaenoics + 6 × hexaenoics)/total FA-concentration;
3. The peroxidation index (PI), showing FA’s susceptibility to peroxidation. Calculated as follows: (0.025 × monoenoics + 1 × dienoics + 2 × trienoics + 4 × tetraenoics + 6 × pentaenoics + 8 × hexaenoics)/total FA-concentration.

Subsequently, we compared the results of these index tests to the patterns that emerged from the interpretation of the differences between patients and controls in the individual FA. For this, we compared the index test results to the individual FA-tests on multiply imputed data, and also constructed the indices from imputed data. In this way, we prevented missing values in the original dataset causing many missing values among the indices, which would have reduced statistical power.

### Statistical Software

We used PASW statistics 18.0 (SPSS, Inc., 2009, Chicago, IL, USA). MI was performed using Amelia II [15], available via the R software package [19].

## Results

### Correlation between Percentages and Concentrations

Table 1 shows the difference between percentages and concentrations (expressed as *r*_absolute–percentual_) for each FA. Correlations ranged from 0.30 for 18:0 to 1.00 for 16:1n-9.

In the second-level analysis, linear regression showed that mean_FA(*i*)_ was associated with *r*(*i*)_absolute–percentual_ (β = −0.685; *t*_(207)_ = −4.882; *P* < 0.001). This indicates that results presented in percentages or concentrations differed more for FA with higher concentrations.

**Fig. 1:**
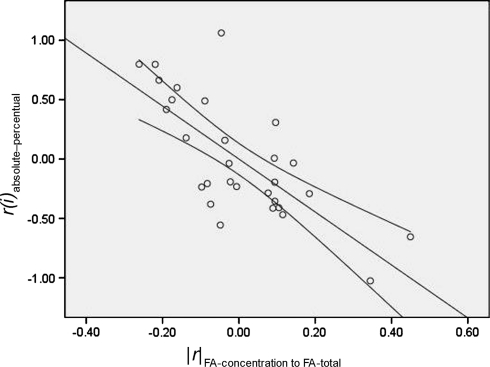
Second level analysis stepwise partial regression plot of the relationship between |*r*|_FA-concentration to FA-total_ [the absolute (non-negative) value of the correlation between the FA-concentration and total FA concentration for an individual subject] with *r*(*i*)_absolute–percentual_ [the correlation between the presentation of a FA as a percentage or as a concentration calculated using Pearson’s *r* (as an indicator of the difference between the two presentational methods)] after Fisher *r*-to-*Z* transformation in an example dataset of 29 FA concentration of 137 recurrently depressed patients and 73 healthy controls. *Lines* represent linear fit and 95 % CI. *FA* fatty acid, *UI* unsaturation index, *CLI* chain length index, *PI* peroxidation index, *MI* multiple imputation

Furthermore, when |*r*|_FA-concentration–FA-total_ was also included in the regression model, it had an independent negative influence on *r*_absolute–percentual_ (β = −0.824; *t*_(207)_ = −5.486; *P* < 0.001; Fig. 1). The influence of mean_FA(*i*)_ on *r*_absolute–percentual_ was no longer significant. This indicates that differences between results presented in percentages and concentrations were significantly greater for those FA that have a stronger correlation with the total FA-concentration, and that this influence explained the effect of high FA concentrations on differences between results presented in percentages or concentrations.

### Handling of Non-detectable Values

In our example dataset, 21 patients and 8 controls had missing FA-results due to technical reasons. The non-detectable percentage ranged from 0 % for 16:0-24:0, 22:5n-3, 22:6n-3, C18:2n-6, 20:3n-6, 22:4n-6, 22:5n-6, 18:1n-7, 18:1n-9 and 24:1n-9, to 60.5 % for 22:2n-6. The mean non-detectable percentage was 11.1 %.

The impact of different methods to handle missing/non-detectable values on example outcomes are demonstrated in Table 1. Compared to results obtained after MI, substitution of non-detectable values with zero resulted in different significance-levels for comparisons between patients and controls. Using zero substitution, the difference between patients and controls in 20:5n-3 was not significant, and differences in 20:3n-6 and 16:1n-9 were less significant. Other FA results were comparable, with lower concentrations for FA with non-detectable values, reflecting the expected bias toward zero.

When non-detectable values were omitted and not used in the analyses, the differences between patients and controls in 18:4n-3, 20:5n-3, 18:3n-6, 20:3n-6 were less or no longer significant, while significant differences in 20:1n-7 and 22:1n-9 emerged and differences in 22:2n-6 could not be tested, all compared to results obtained after MI.

### Using Indices to Describe FA Patterns

The tests on the 29 individual FA after multiple imputation are listed in the right columns of Table 1. First, a Bonferroni correction for multiple comparisons was performed resulting in a corrected α of 0.05/29 = 0.0017. After this correction, differences between patients and controls for 20:5n-3, 20:3n-6, 16:1n-7 and 16:0 were no longer significant. Other differences remained significant, with lower concentrations in patients for 22:5n-3, 22:6n-3, 20:3n-6, 20:4n-6, 22:4n-6, 22:5n-6, 14:1n-5, 16:1n-9, 24:1n-9, 20:0, 22:0 and 24:0. Concentrations of 18:4n-3 18:3n-6, 22:2n-6, 16:1n-7, and 16:0 were higher in patients compared to controls. In analogy to our previous interpretations [10], these results fitted with patterns of reduced chain length, unsaturation and peroxidation for FA of the patients.

After data-reduction using the CLI, UI, and PI, differences between patients and controls were calculated (Table 2). The mean FA values for patients were less unsaturated (*P* ∼ 1.2 × 10^−18^; Cohen’s *d* = 2.35), shorter (*P* ∼ 7.1 × 10^−19^; Cohen’s *d* = 1.46), and less peroxidizable (*P* ∼ 4.0 × 10^−15^; Cohen’s *d* = 1.83).

**Table 2:** Mean chain length, unsaturation and peroxidation indices compared between recurrently depressed patients and controls

	Patients	SEM	Controls	SEM	*t*	df	*P* value
Chain length index	18.32	0.0181	18.55	0.0119	10.96	226.5	∼7.1 × 10^−19^
Unsaturation index	1.29	0.0068	1.39	0.0059	11.14	90.21	∼1.2 × 10^−18^
Peroxidation index	1.10	0.0093	1.22	0.0090	9.241	101.5	∼4.0 × 10^−15^

All differences were calculated using independent Student's *t* test on indices of 137 recurrently depressed patients and 73 controls

*SEM* standard error of the mean, *df* degrees of freedom

When comparing the index results to the results of the multiple individual FA-tests, pattern outcomes were similar, with reduced chain length, unsaturation and peroxidation for FA of patients. Using indices resulted in fewer tests, but provided no information on differences in individual FA concentrations between patients and controls.

## Discussion

Our results indicate that: (1) presentation of FA in either percentages or concentrations yields different results, particularly for those FA with a stronger correlation with the total FA-concentration, (2) differences in the approach used for non-detectable/missing values influence significance-levels of outcomes of FA-analysis, and (3) the use of the CLI, UI and PI showed differences between patients and controls in FA-patterns, in agreement with interpretations from individual FA-tests.

Differences between data presented in concentrations and percentages imply that these methods are not simply interchangeable. Moreover, differences between percentages and concentrations depended on individual FA-characteristics (|*r*|_FA-concentration to FA-total_). This dependency could inflict differential biases in individual FA results. Therefore, our findings emphasize the importance of a hypothesis driven choice of which method to use. Percentages could be used as a measure of the relative importance of a FA set against the total FA concentration; while absolute concentrations could be used for the measurement of a FA itself, independent of the concentration of other FA [3–5, 20].

The appropriate method of presentation could theoretically differ for each research question [21]. For example, concentrations could be most useful to distinguish depressed patients from controls, while percentages might predict disease progression. Therefore, the appropriate presentation method may depend upon which presentation is more (patho)physiologically to the research question under investigation. However, the dearth of research comparing both approaches so far, may—at present—hamper the formation of a hypothesis about which method be more (patho)physiologically relevant. If so, comparison of both methods of presentation could provide a guideline for future research.

Our results show that the way non-detectable/missing values are handled could potentially bias results, because significance levels of differences in example outcomes differed depending on which method was used. However, it should be noted that not only significances of differences, but also magnitudes of differences determine the bias introduced. Nevertheless, if non-detects occur, knowledge of the way they were handled, and discussion of any possible bias that may be inflicted as such, could prevent interpretation errors. Because other research fields already showed superiority of MI compared to other ways of handling non-detectable/missing values [6], this may soon be adapted as the preferred method to handle missing/non-detectable FA-concentrations as well.

By applying data reduction using indices—the CLI, UI and PI—we tested differences between patients and controls in FA-patterns. Index results were similar to the interpretation of the multiple tests on individual FA [10]. This suggests that indices could provide meaningful data-reduction in FA research. Furthermore, from a statistical viewpoint, the use of indices enabled us to test pattern hypotheses more efficiently by using only one outcome variable (CLI, UI or PI), instead of tests of many individual FA. This precluded the need for correction for multiple tests. In our example dataset this was not of specific analytical concern, because differences in individual FA-tests were large and mostly survived the Bonferroni correction. Nevertheless, this advantage may be beneficial in smaller samples or in diseased populations with smaller differences compared with controls. In addition, the indices facilitated quantitative testing of pattern hypotheses, in contrast to the qualitative interpretation of the individual FA tests. The disadvantage of integrating information on multiple FA-concentrations in one index, could be that it might undesirably simplify the underlying complexity of FA-metabolism. In such situations the relevance of an individual FA could be obscured, because differences in individual FA are not tested.

Whether indices should be used in FA-research seems to depend on the hypothesis under investigation. If FA are analyzed in order to test a pattern [e.g. membrane fluidity; unsaturation or peroxidizability; estimated enzyme activity; (inflammation regulating) FA ratios], indices could be used to first test this general pattern hypothesis. Subsequently, based on the index results, new specific hypotheses concerning selected individual FA could be tested. This would reduce the risk for type-I errors, or the need for strict correction for multiple testing [8]. A recent example of the possible usefulness of applying indices is the observation of bimodal distributions of FA unsaturation and chain length patterns in recurrently depressed patients [22]. However, if FA are analyzed to test a hypothesis concerning a specific FA (e.g. EPA), indices have no use, and should not be tested additionally since this would only increase the problem of multiple testing. Future studies are needed to further clarify the applicability of indices in FA-research.

Some additional limitations should be noted. The examples of the possible influences of the presented statistical methodological issues have been presented on the basis of only one dataset. However, although the size of the biasing effects may differ between different datasets, the basic principles of the issues addressed concern analysis of FA data in general. Second, our example dataset has a moderate sample size when compared to epidemiological studies. This could have influenced the stability of correlation coefficients, and therefore the results. Third, the data presented only concern these three statistical methodological issues, and do not investigate other important factors that may also influence results, e.g. chemical analytical methods, and the nature of the sample (tissue, cell type, lipid fraction, e.g. cholesteryl esters, triacylglycerol, phospholipids) [4]. Finally, because differences in outcome measures were large in our example dataset, the disadvantages of multiple testing—and thereby the advantages of data reduction—could not be clearly exemplified and remain to be further explored in different datasets.

Nevertheless, our study addresses recurrent basic issues in practical FA research. Using a second-level analysis we were able to quantitatively demonstrate the consequences of the various methods of presentation. In addition, we suggested a novel way to handle non-detectable FA-values, using MI. Finally, we showed, to our knowledge for the first time, that indices could be used to delineate differences in FA patterns between depressed patients and controls.

In conclusion, a hypothesis-based choice of the method of FA-presentation (percentages or concentrations) could prevent bias in future FA-research. If it is not clear which method is preferable a priori, comparison of both methods could guide subsequent investigations. Furthermore, MI might prevent bias potentially inducible by missing/non-detectable values. Finally, indices could assist theory based data-reduction, thereby preventing type-I errors associated with multiple testing. Awareness and cautious handling of these statistical methodological issues in future FA-research may further improve interpretation of FA-analyses, and thereby deepen the understanding of the roles FA play in health and disease.
